# A Fatal Case of Opioid Intoxication After Raw Poppy Plant Ingestion

**DOI:** 10.7759/cureus.13176

**Published:** 2021-02-06

**Authors:** Gurkan Bozan, Hasan Bora Ulukapi, Ummuhan Oncul, Serkan Levent, Ener C Dinleyici

**Affiliations:** 1 Pediatric Intensive Care Unit, Eskisehir Osmangazi University Faculty of Medicine, Eskisehir, TUR; 2 Department of Pediatrics, Eskisehir Osmangazi University Faculty of Medicine, Eskisehir, TUR; 3 Department of Pharmaceutical Chemistry, Anadolu University Faculty of Pharmacy, Eskisehir, TUR

**Keywords:** naloxone, opioids, papaver somniferum, plant alkaloids, poppy

## Abstract

*Papaver somniferum *contains many opioids and is frequently used in agriculture. Both the intoxication and the withdrawal of opioids have a wide range of symptoms such as coma, depressed respiration and agitation. Here, a fatal case of opioid intoxication will be presented. A four-year-old female patient was admitted to the pediatric intensive care unit after ingesting raw poppy plants. She had shallow respiration, tachycardia, hypertension and muscle cramps. A high plasma opioid level was measured and bolus intravenous naloxone was administered which resulted in a brief gain of consciousness. She was intubated after a sudden respiratory depression and loss of consciousness 10 hours later. Naloxone infusion was started and continued for two days. She developed disseminated intravascular coagulation and was lost on day twelve. Raw plant ingestion proves difficult to treat since there is less information about the ingredients. Having no consensus on naloxone dosage and intrinsic complications such as hypo- and hypertension, redistribution, rhabdomyolysis and dysmotility disrupts naloxone administration. Ingestion of opioids as plants brings out different complications for the treatment course while deciding on naloxone dosage proves opioid intoxication difficult to treat.

## Introduction

Since poppy seed has an economic value as an industrial plant, it is frequently grown in Turkey as well as in different regions of the world. *Papaver somniferum* subsp. *anatolicum* var. *nigrum* (blind poppy) is the variant that is mostly used in agriculture. It consists of 99 kinds of alkaloids [1]. Ones that are found in the highest concentrations are morphine, codeine, tebaine, noscapine, and papaverin [2]. Alkaloid concentrations change according to harvest timing. Phenantrene alkaloids in poppy seeds can cause severe addiction. While opioid intoxication causes bradycardia, coma, decreased gastrointestinal system (GIS) motility, depressed mental state, depressed respiration, hypotension, hypothermia and myosis, opioid withdrawal may present with agitation, diarrhea, diaphoresis, hypertension, cramps, midriasis, piloerection, tachycardia, tachypnea, vomiting, and yawning [3]. Although naloxone has been used as a specific antidote for opioid intoxication since 1960, suggested dosing and administration routes vary widely in different sources [4]. Here, a fatal case of opioid intoxication will be presented.

## Case presentation

**Figure 1:**
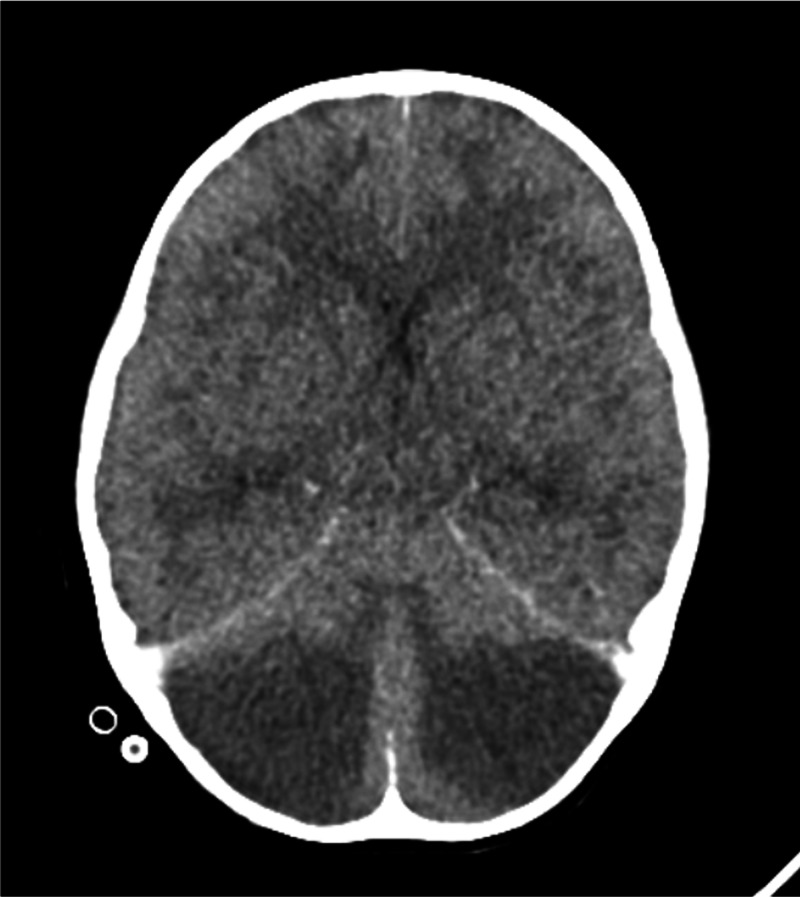
Brain computerized tomography (CT) showed edema in bilateral infratentorial region in both hemispheres.

A four-year-old female patient was brought to the pediatric emergency department after her parents could not wake her up. Her family were occupied as poppy seed farm workers in Afyon province of Turkey and reported that they all ate raw poppy plants approximately 12 hours prior. The patient was unconscious and her Glasgow Coma Score (GCS) was 5, her respiration was shallow and she had myoclonus. She was quickly admitted to the Pediatric Intensive Care Unit. Non-invasive mechanical ventilation was started. She had tachycardia and hypertension. Neurological examination revealed no response to painful stimuli and hyperactive deep tendon reflexes. PRISM III score was 20 and the expected mortality rate was 29.8%. Intravenous (IV) naloxone at 0.1 mg/kg/dose was administered after plasma opioid level was reported to be 1196 ng/mL. She had a brief gain of consciousness; GCS was 7. Large amounts of poppy seed were extracted during gastric lavage done within one hour of admission. Lavage was continued until clear irrigation water was obtained. The patient was washed in case of accompanying organophosphate intoxication. Venous blood gas examination showed a lactate level of 4.5 mmol/L. Plasma creatinine kinase (CK) was 2263 IU/L. Alkaline fluid at 3000 ml/m2/day was administered in order to prevent the effects of rhabdomyolysis. Hemodialysis through a catheter inserted into the right jugular vein was performed after consideration of volume load and urine output since the control CK level was 9194 IU/L in order to prevent renal damage due to rhabdomyolysis. IV N-acetyl cysteine (NAC) infusion was administered after cardiac markers were found to be elevated. A sudden depression of respiration developed at the 10th hour of her care, and was intubated and supported via invasive mechanic ventilation in synchronized intermittent mandatory ventilation (SIMV) pressure control mode. Cefotaxime and teicoplanin antibiotherapy was initiated after an infiltration in the upper zone of the right lung was noticed in the posteroanterior chest radiograph taken to assess the position of the endotracheal tube. IV of 0.0025 mg/kg/hour naloxone infusion was initiated as the patient lost her consciousness again (GCS: 3). After one dose of 20 ml/kg bolus saline, IV 5 mcg/kg/min dopamine infusion was initiated since the patient was hypotensive, the dose was gradually increased, dobutamine and adrenaline were added. While the initial vasoactive-inotropic score was 0, it increased to 5 at the 12th hour, and to a maximum of 60 at the 24th hour. Inotropic agents were gradually stopped after hypertension developed under naloxone infusion. IV hydration was decreased to 2000 ml/m2/day. Naloxone infusion was gradually stopped after plasma opiate levels of <70 ng/mL were detected on the second day of her care. IV 0.3 mcg/kg/day desmopressin acetate was administered after detecting that urine output was 9.6 ml/kg/hour and plasma sodium (Na) level was 169 mEq/L and further investigations (urine Na, plasma and urine osmolarity) were done and a diagnosis of diabetes insipitus was made. Brain computerized tomography (CT) showed edema in bilateral infratentorial region in both cerebellar hemispheres (Figure 1).

**Figure 2:**
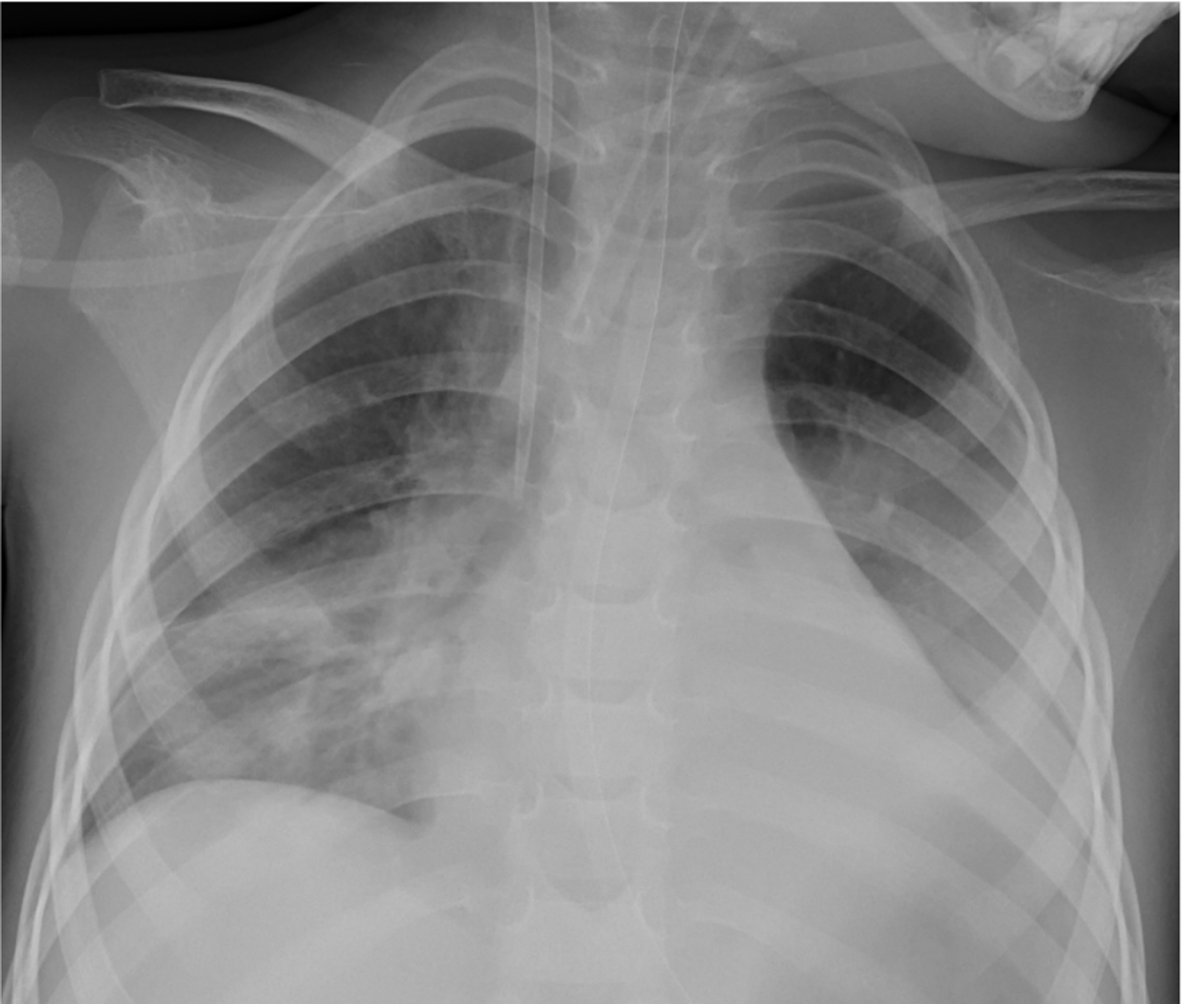
Chest X-ray showed new infiltrations.

IV 1 mg/kg/day dexamethasone treatment was initiated. Total parenteral nutrition was administered since the patient did not improve after two days of IV hydration in hopes for regression of respiratory depression and neurological symptoms after cessation of naloxone infusion. On the sixth day of her care, gastric residue was detected after initiation of enteral feeding via a nasogastric tube. The residue still had poppy seeds in its content. Gastric lavage was repeated. High amounts of poppy seed were extracted. Control opiate level was 78 ng/mL. Daily gastric lavage was continued. Near-infrared spectroscopy (NIRS) and ambulatory electroencephalogram (EEG) monitorization was done. NIRS was between 56-60%. Electrical brain activity was severely reduced in the EEG. Permissive hypercapnia was achieved. A control brain CT that was performed in order to assess progression of brain edema showed continuation of edema. Mannitol treatment was initiated. A d-dimer level of >34 ng/dL was reported while prothrombin time and fibrinogen levels were normal. The patient’s disseminated intravascular coagulation score was 5 and fresh frozen plasma was transfused. No defecation was observed for three consecutive days and a rectal enema was applied. High amounts of poppy seed were observed in the feces on the eighth day of her care. Her general condition had worsened on the 12th day of her care while thrombocytopenia developed and new infiltrations were observed in her chest x-ray (Figure 2).

**Figure 3:**
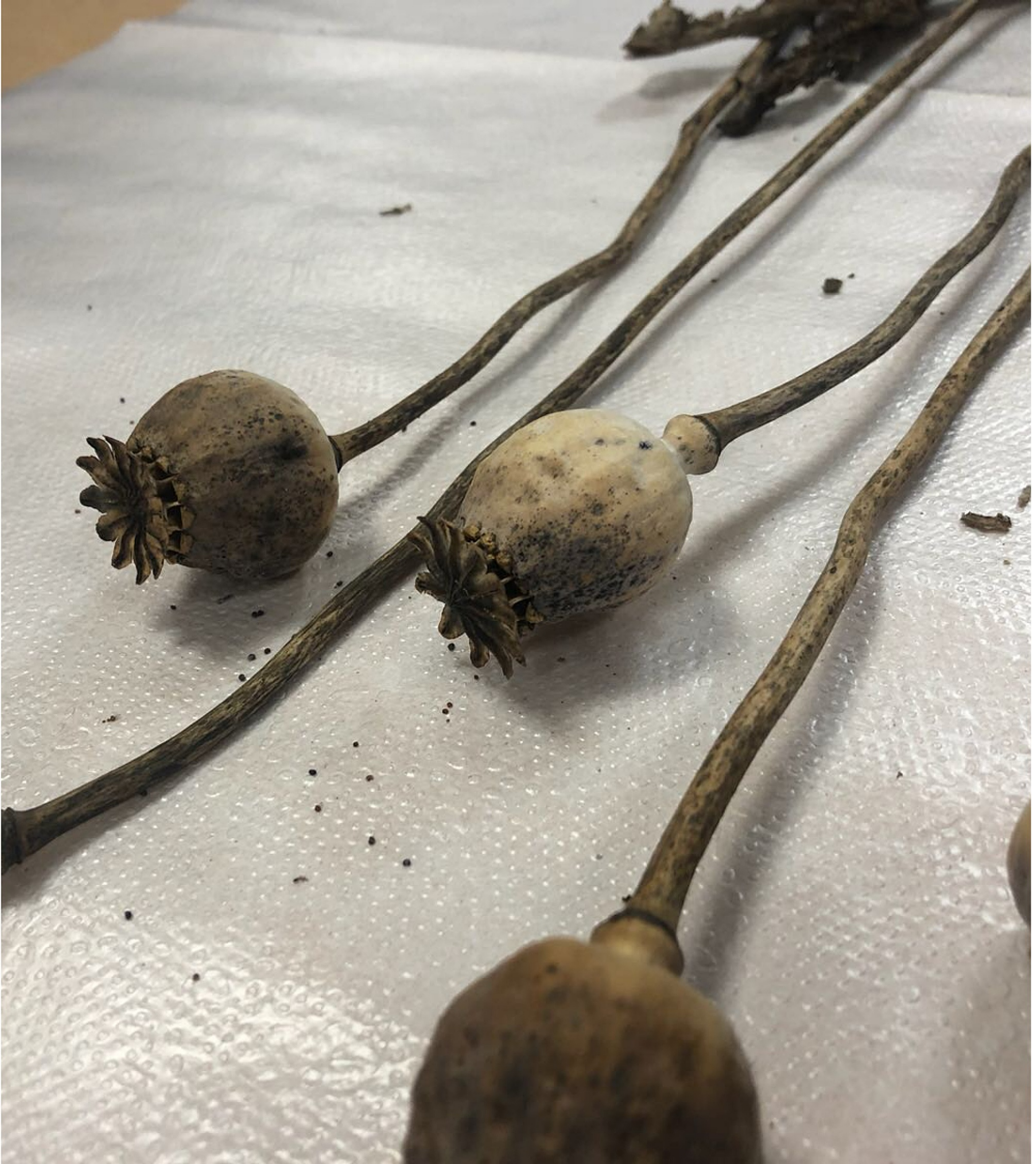
The poppy plant that the patient was thought to have ingested

Her antibiotherapy was revised as caspofungin, meropenem and linezolid. Hypertension resistant to furosemide, captopril and amlodipine was observed. IV esmolol infusion was initiated. Subsequently, hypotension followed. Esmolol infusion was gradually decreased and stopped. Inotropic agents were administered as the patient was hypotensive and the electrocardiogram showed widespread ST depression. Control plasma opioid level was 20 ng/dL. The patient was bleeding through the endotracheal tube and quickly had a cardiac arrest. She did not respond to cardiopulmonary resuscitation (CPR) and died. The poppy plant (Figure 3) that the patient was thought to have ingested was analyzed and reported to consist of unusually high levels (never before measured as high by the Anatolian University Farmaceutic Toxicology Department) of morphine, codeine, acetylhydrocodeine, papaverine and atropine alkaloids (Table 1). 

**Table 1:** Alkaloids found in the ingested poppy plant.

Compounds	Mol. Formula	Expected [M+H]^+^	Measured [M+H]^+^	Amount (%)
Morphine	C_17_H_19_NO_3_	286.1438	286.1435	55.1
Codeine	C_18_H_21_NO_3_	300.1594	300.1606	9.4
Thebaine	C_19_H_21_NO_3_	312.1594	312.1599	16.3
Papaverine	C_20_H_21_NO_4_	340.1543	340.1555	3.9
Acetyldihydroxycodeine	C_20_H_25_NO_4_	344.1856	344.1859	7.0
Salutaridinol	C_19_H_23_NO_4_	330.1700	330.1701	6.8
Isopavine	C_20_H_23_NO_4_	342.1700	342.1709	1.5

## Discussion

In developed countries, opioid intoxication is generally due to recreational use or suicide and such events usually allow for information on the ingredients and dosage of the agents. In Turkey, in the socioculturally low regions, plants that consist of these alkaloids are usually ingested as a recreational activity. Our patient's family were seasonal agriculture workers in Afyon province that were occupied in a poppy seed farm. They commented as having eaten raw poppy many times before, which is not unusual as the patient history in our clinic. No symptoms other than unconsciousness were reported by the family, probably because the whole family was sleeping and/or under the effect of opium themselves. That is why we mainly focused on the diagnosis of opium poisoning. The analysis of the suspected plant that the patient had eaten consisted of five opioids in never-before-seen high amounts, but sadly, this information was available after the loss of our patient. Raw plant ingestion thus proves difficult to treat due to little information on the ingredients of the plant. On the other hand, there is no consensus on the dosage of naloxone infusion with references varying by 13-fold. While algorithms are trying to be developed to decide on the dosage [5], complications during patient care disrupt these algorithms [6]. Hypertension as a side effect of naloxone, opioid redistribution from adipose tissue [7], difficulty removing opioids due to GIS motility dysfunction [8] and rhabdomyolysis [9] are some of these complications. Our patient needed an increase in naloxone infusion dose due to frequent exposure to opioids but hypertension did not allow for higher doses. Even though opioid levels returned to normal, her consciousness was still absent. Due to GIS motility dysfunction, even though defecation was observed after rectal enema and daily gastric lavage removed large amounts of poppy seeds, large amounts of poppy seeds were still apparent during CPR.

Pseudocholinesterase levels were not measured since it is not readily available in our lab, but we believe that the cause of lengthened effects of opium was due to disturbed gastric motility rather than pseudocholinesterase deficiency. No evidence of GIS obstruction was seen, unlike the case of Schuppener et al. [8]. Total gastrointestinal decontamination techniques were not applied because, in patients with decreased motility, no clinical evidence was reported to be helpful [10]. NAC infusion after the rise of cardiac markers was started because NAC has been shown to have an antioxidant effect since it increases glutathione production in heart cells by providing cysteine, and scavenges several reactive oxygen species. Its beneficial effect on ischemic heart injury, cardiac failure and remodeling have been shown [11]. Hypernatremia and diabetes insipitus developed in our patient but no similar case has been reported before. Rhabdomyolysis is a common complication of opioid intoxication [9] and was also seen in our patient. Hydration with alkaline fluids and hemodialysis were efficient in preventing its effects. This case was managed before the 2020 Surviving Sepsis Campaign International Guidelines [12] were published; therefore inotropes were initiated with dopamine, though adrenaline infusion followed quickly. Although opioid intoxication is not only seen in people with opioid addiction, current studies concerning the pediatric population on opioid intoxication mainly focus on opioid overdose in adolescents within the context of the opioid epidemic [13] or withdrawal management in neonatal abstinence syndrome [14].

## Conclusions

Opioid intoxication can also occur with raw ingestion of the poppy plant or seed or as a tea. Ingestion of opioids as a plant instead of the drug form brings out many different complications for the course of treatment and requires specific research on management. Lack of consensus in naloxone treatment and dealing with side effects in addition to complications of intoxication all contribute to difficulty in treatment. Effects of opioids such as GIS motility dysfunction and rhabdomyolysis should not be forgotten beside the more drastic effects like respiratory depression and change in consciousness and should be researched. It should be remembered that opioid intoxication can be fatal.
